# Coronavirus Susceptibility to the Antiviral Remdesivir (GS-5734) Is Mediated by the Viral Polymerase and the Proofreading Exoribonuclease

**DOI:** 10.1128/mBio.00221-18

**Published:** 2018-03-06

**Authors:** Maria L. Agostini, Erica L. Andres, Amy C. Sims, Rachel L. Graham, Timothy P. Sheahan, Xiaotao Lu, Everett Clinton Smith, James Brett Case, Joy Y. Feng, Robert Jordan, Adrian S. Ray, Tomas Cihlar, Dustin Siegel, Richard L. Mackman, Michael O. Clarke, Ralph S. Baric, Mark R. Denison

**Affiliations:** a Department of Pathology, Microbiology, and Immunology, Vanderbilt University Medical Center, Nashville, Tennessee, USA; b Department of Pediatrics, Vanderbilt University Medical Center, Nashville, Tennessee, USA; c Department of Epidemiology, University of North Carolina at Chapel Hill, Chapel Hill, North Carolina, USA; d Department of Biology, the University of the South, Sewanee, Tennessee, USA; e Gilead Sciences, Inc., Foster City, California, USA; NIAID, NIH

**Keywords:** RNA polymerases, SARS-CoV, antiviral agents, antiviral resistance, coronavirus, nucleoside analogs, pandemic

## Abstract

Emerging coronaviruses (CoVs) cause severe disease in humans, but no approved therapeutics are available. The CoV nsp14 exoribonuclease (ExoN) has complicated development of antiviral nucleosides due to its proofreading activity. We recently reported that the nucleoside analogue GS-5734 (remdesivir) potently inhibits human and zoonotic CoVs *in vitro* and in a severe acute respiratory syndrome coronavirus (SARS-CoV) mouse model. However, studies with GS-5734 have not reported resistance associated with GS-5734, nor do we understand the action of GS-5734 in wild-type (WT) proofreading CoVs. Here, we show that GS-5734 inhibits murine hepatitis virus (MHV) with similar 50% effective concentration values (EC_50_) as SARS-CoV and Middle East respiratory syndrome coronavirus (MERS-CoV). Passage of WT MHV in the presence of the GS-5734 parent nucleoside selected two mutations in the nsp12 polymerase at residues conserved across all CoVs that conferred up to 5.6-fold resistance to GS-5734, as determined by EC_50_. The resistant viruses were unable to compete with WT in direct coinfection passage in the absence of GS-5734. Introduction of the MHV resistance mutations into SARS-CoV resulted in the same *in vitro* resistance phenotype and attenuated SARS-CoV pathogenesis in a mouse model. Finally, we demonstrate that an MHV mutant lacking ExoN proofreading was significantly more sensitive to GS-5734. Combined, the results indicate that GS-5734 interferes with the nsp12 polymerase even in the setting of intact ExoN proofreading activity and that resistance can be overcome with increased, nontoxic concentrations of GS-5734, further supporting the development of GS-5734 as a broad-spectrum therapeutic to protect against contemporary and emerging CoVs.

## IMPORTANCE

Coronaviruses (CoVs) cause severe human infections, but there are no approved antivirals to treat these infections. Development of nucleoside-based therapeutics for CoV infections has been hampered by the presence of a proofreading exoribonuclease. Here, we expand the known efficacy of the nucleotide prodrug remdesivir (GS-5734) to include a group β-2a CoV. Further, GS-5734 potently inhibits CoVs with intact proofreading. Following selection with the GS-5734 parent nucleoside, 2 amino acid substitutions in the nsp12 polymerase at residues that are identical across CoVs provide low-level resistance to GS-5734. The resistance mutations decrease viral fitness of MHV *in vitro* and attenuate pathogenesis in a SARS-CoV animal model of infection. Together, these studies define the target of GS-5734 activity and demonstrate that resistance is difficult to select, only partial, and impairs fitness and virulence of MHV and SARS-CoV, supporting further development of GS-5734 as a potential effective pan-CoV antiviral.

## INTRODUCTION

Coronaviruses (CoVs) are positive-sense, single-stranded RNA viruses that infect a wide range of animal hosts. In humans, CoVs were recognized as typically causing colds and pneumonia until the emergence of severe acute respiratory syndrome coronavirus (SARS-CoV) in 2002 and Middle East respiratory syndrome coronavirus (MERS-CoV) in 2012 from zoonotic sources (1, 2). Although the SARS epidemic was controlled by public health measures within a year of its emergence, the virus spread to over 30 countries and was associated with a 10% mortality rate (3). Efforts to treat SARS patients with existing antivirals did not conclusively provide a clinical benefit and may have even worsened disease (4–7). MERS-CoV continues to circulate in the Middle East, with a case fatality rate approaching 40% (http://www.who.int/emergencies/mers-cov/en/). Currently, there are no FDA-approved antivirals or vaccines for the treatment and prevention of MERS-CoV infection. Supportive care and prevention of complications constitute the current standard of treatment for patients, emphasizing the need for direct-acting antivirals (8, 9). Furthermore, SARS- and MERS-like bat CoVs circulate in nature, can replicate efficiently in primary human airway cells, and use the same cellular receptors for entry as human CoVs (10–13). The imminent threat of human emergence underscores the need for broadly active antivirals to combat any CoV that may emerge.

Nucleoside analogues commonly target viral replication, particularly the viral DNA or RNA polymerase (14), and have succeeded clinically in treating multiple viral infections (15). However, identification and development of antiviral nucleosides against coronaviruses have been hampered by the presence of the unique CoV proofreading 3′-5′ exoribonuclease (ExoN) (16–18). While nucleoside analogues such as BCX4430 inhibit CoVs (19), several previously tested nucleoside analogues have been incapable of potently inhibiting CoV replication, and others have demonstrated poor selectivity indexes (20, 21). We have shown that CoV resistance to the mutagens 5-fluorouracil (5-FU) and ribavirin (RBV) *in vitro* is attributed to their removal by the proofreading ExoN (22), supporting the hypothesis that an effective nucleoside analogue must evade proofreading to successfully interfere with CoV RNA synthesis.

**FIG 1:**
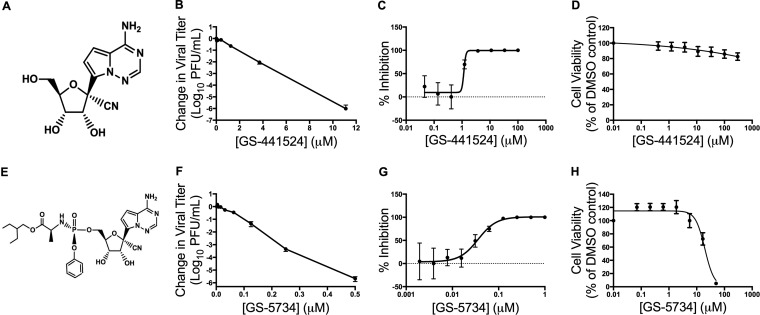
GS-441524 and GS-5734 inhibit MHV with minimal cytotoxicity. (A) GS-441524 is a 1′-cyano 4-aza-7,9-dideazaadenosine C-adenosine nucleoside analogue. (B) Change in viral titer of MHV compared to vehicle control after treatment with GS-441524. The data represent the results from 2 independent experiments, each with 3 replicates. Error bars represent standard error of the mean (SEM). (C) Viral titer data from panel B presented as the percentage of uninhibited control. The EC_50_ of GS-441524 was calculated to be 1.1 μM. (D) Cell viability normalized to the vehicle control after treatment with GS-441524. The data represent the results from 3 independent experiments, each with 3 replicates. Error bars represent SEM. (E) GS-5734 is a monophosphoramidate prodrug of GS-441524. (F) Change in viral titer of MHV compared to vehicle control after treatment with GS-5734. The data represent the results from 4 independent experiments, each with 3 replicates. Error bars represent SEM. (G) Viral titer data from panel F presented as the percentage of uninhibited control. The EC_50_ of GS-5734 was calculated to be 0.03 μM. (H) Cell viability normalized to vehicle control after treatment with GS-5734. The data represent the results from 3 independent experiments, each with 3 replicates. Error bars represent SEM.

We recently reported that GS-5734, the monophosphoramidate prodrug of the C-adenosine nucleoside analogue GS-441524 (Fig. 1A), inhibits SARS-CoV, MERS-CoV, and bat CoV strains that are capable of replicating in primary human airway epithelial cells and mediate entry using human CoV receptors (23–25). GS-5734 also demonstrates both prophylactic and therapeutic efficacy against SARS-CoV disease in a mouse model (23). However, the study was not designed to define, nor did it report, potential pathways and implications of resistance for virus fitness and virulence. Further, studies demonstrating the efficacy of GS-5734 against CoVs and other viruses, including Ebolavirus, have not described resistance mutations. Using the model β-coronavirus murine hepatitis virus (MHV), we here demonstrate that GS-5734 dramatically inhibits viral replication and viral RNA synthesis in wild-type (WT) virus, while an nsp14 ExoN(−) mutant lacking proofreading demonstrates increased susceptibility to GS-5734. Passage of WT MHV with the GS-5734 parent nucleoside GS-441524 resulted in phenotypic resistance associated with two nonsynonymous mutations in the predicted fingers domain of the nsp12 RNA-dependent RNA polymerase (F476L and V553L). The engineered mutations in the MHV cloned background closely recapitulated the partial resistance phenotype and restored RNA levels in the presence of GS-5734. However, resistant viruses could not compete with WT MHV during *in vitro* coinfection passage in the absence of GS-5734. Introduction of homologous substitutions in mouse-adapted SARS-CoV conferred resistance to GS-5734 similar to that seen in MHV but also attenuated *in vivo* pathogenesis of SARS-CoV in a mouse model. Overall, our results are consistent with an RNA-dependent RNA polymerase (RdRp)-mediated mechanism of potent CoV inhibition by GS-5734, even in the setting of intact ExoN-mediated proofreading.

## RESULTS

### GS-441524 and GS-5734 inhibit MHV replication.

GS-441524, a 1′-cyano 4-aza-7,9-dideazaadenosine C-nucleoside (Fig. 1A), has been shown to inhibit multiple virus families *in vitro* (24, 26). To determine if GS-441524 inhibited the model β-2a CoV, murine hepatitis virus (MHV), we infected delayed brain tumor (DBT) cells with MHV and treated them with increasing concentrations of drug. We observed a dose-dependent reduction in viral titer with up to a 6-log_10_ decrease at 11.1 μM GS-441524 (Fig. 1B). The half-maximum effective concentration (EC_50_) value resulting from GS-441524 treatment was 1.1 μM (Fig. 1C). We observed minimal detectable cytotoxicity within the tested range, with the concentration resulting in 50% cytotoxicity (CC_50_) >300 μM (Fig. 1D). This resulted in a selectivity index (CC_50_/EC_50_) of >250. Having demonstrated the inhibition of MHV by GS-441524, we next tested its monophosphoramidate prodrug GS-5734 (Fig. 1E). Treatment with increasing concentrations of GS-5734 resulted in up to a 6-log_10_ decrease in viral titer, and virus was undetectable by plaque assay at concentrations above 0.5 μM GS-5734 (Fig. 1F). GS-5734 inhibited MHV more potently than GS-441524, with a GS-5734 EC_50_ of 0.03 μM (Fig. 1G), consistent with higher cellular permeability and more efficient metabolism of the prodrug into the active nucleoside triphosphate by bypassing the rate-limiting first phosphorylation step (27, 28). We also observed minimal cytotoxicity at concentrations required for antiviral activity of GS-5734, in line with previously reported extensive cytotoxicity studies in relevant human cell types (27), with a CC_50_ value of 39 μM (Fig. 1H), resulting in a selectivity index of >1,000. These results expand the breadth of GS-441524 and GS-5734 inhibition of CoVs to include the β-2a model CoV MHV.

### GS-441524 and GS-5734 potently inhibit SARS-CoV and MERS-CoV in HAE cells.

**FIG 2:**
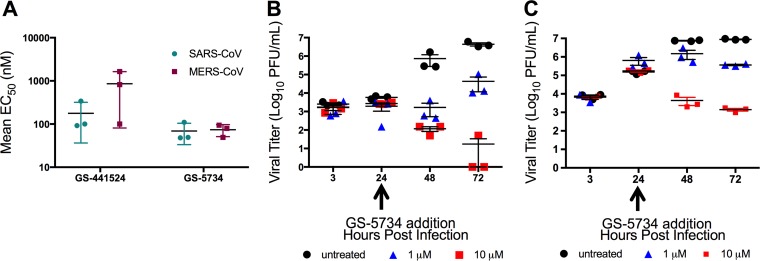
Antiviral activity of GS-441524 and GS-5734 and modeled therapeutic efficacy of GS-5734 against SARS-CoV and MERS-CoV in HAE cultures. (A) Mean EC_50_ values of SARS-CoV and MERS-CoV-infected HAE cultures from three different patient isolates treated with GS-441524 or GS-5734. (B) Viral titers of SARS-CoV-infected HAE cultures when treated with various doses of GS-5734 24 h postinfection. (C) Viral titers of MERS-CoV-infected HAE cultures when treated with various doses of GS-5734 24 h postinfection.

Primary human airway epithelial cell (HAE) cultures are among the most clinically relevant *in vitro* models of the lung, recapitulating the cellular complexity and physiology of the epithelium in the human conducting airway (29). Previous results have demonstrated that GS-5734 inhibits the viral titer of multiple CoVs in this model, but did not assess the potency or the effect of delaying treatment with the compound. Thus, we determined the EC_50_ values after treatment with GS-441524 and GS-5734 in SARS-CoV- and MERS-CoV-infected HAE cultures. Mean EC_50_ values for both viruses were approximately 0.86 μM for GS-441524 and 0.074 μM for GS-5734 (Fig. 2A). Further, delaying addition of GS-5734 until 24 hours (h) postinfection resulted in decreased viral titer in HAE cultures for both SARS-CoV (Fig. 2B) and MERS-CoV (Fig. 2C) at 48 and 72 h postinfection. No measurable cellular toxicity was observed in HAE cultures for either compound (Table 1). These results demonstrate a similar high potency of GS-5734 across divergent CoVs, supporting the utility of the model MHV system to study GS-5734 inhibition and resistance.

**TABLE 1:** EC_50_ and CC_50_ values of GS-441524 or GS-5734 in MERS-CoV- or SARS-CoV-infected HAE cultures^a^

Virus	GS-441524		GS-5734	
	EC_50_ (μM)	CC_50_ (μM)	EC_50_ (μM)	CC_50_ (μM)
MERS	0.86 ± 0.78	>100	0.074 ± 0.023	>10
SARS	0.18 ± 0.14	>100	0.069 ± 0.036	>10

a Values represent the average (mean ± SD) from HAE cultures from at least three donors.

### GS-5734 acts at early times postinfection to decrease viral RNA levels.

**FIG 3:**
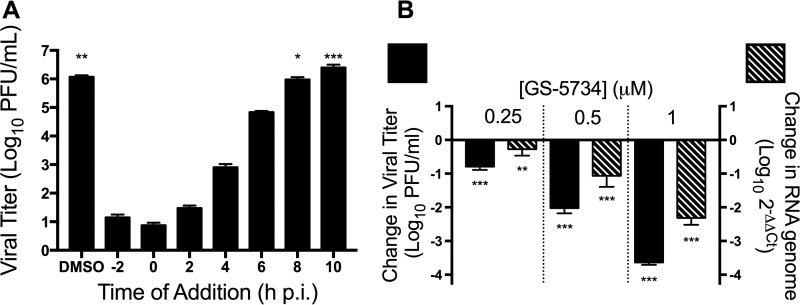
GS-5734 acts at early times postinfection to decrease viral RNA levels. (A) MHV viral titer after single-cycle infection and treatment with 2 μM GS-5734 at the indicated times postinfection. The data represent the results from 2 independent experiments, each with 3 replicates. Error bars represent SEM. Statistical significance compared to addition of GS-5734 at 0 h postinfection (p.i.) was determined by one-way analysis of variance (ANOVA) with Dunnett’s *post hoc* test for multiple comparisons and is denoted by asterisks: *, *P* < 0.05; **, *P* < 0.01; ***, *P* < 0.001. (B) Change in viral titer (black bars) and viral RNA levels (hatched bars) normalized to vehicle control 10 h postinfection after treatment with GS-5734. The data represent the results from 2 independent experiments, each with 3 replicates. Error bars represent SEM. Statistical significance compared to DMSO-treated samples was determined by one-way ANOVA with Dunnett’s *post hoc* test for multiple comparisons and is denoted by asterisks: **, *P* < 0.01; ***, *P* < 0.001.

The predicted mechanism of action of GS-5734 is through incorporation of the active triphosphate into viral RNA (27). We therefore tested the hypothesis that GS-5734 would inhibit CoVs at early steps in replication by inhibiting viral RNA synthesis. To determine which stage in the viral replication cycle GS-5734 inhibited CoVs, we infected cells with MHV at a multiplicity of infection (MOI) of 1 PFU/cell, which with MHV results in a single-cycle infection, and treated them with 2 μM GS-5734 at 2-h intervals from 2 h preinfection to 10 h postinfection. We observed maximal inhibition when GS-5734 was added between 2 h preinfection and 2 h postinfection. Less inhibition was detected when GS-5734 was added between 4 and 6 h postinfection, and no inhibition was observed when GS-5734 was added after 8 h postinfection (Fig. 3A). These results demonstrate that GS-5734 inhibits CoVs at early steps during infection. Because viral RNA is synthesized early in infection and GS-5734 is implicated in inhibiting viral RNA synthesis (25, 30, 31), we next determined the cellular level of viral RNA by real-time quantitative PCR (qPCR) after treatment with GS-5734. Treatment with increasing concentrations of GS-5734 resulted in decreased viral RNA levels that correlated with the decrease in titer we observed (Fig. 3B). These results suggest that GS-5734 inhibits CoVs early after infection by interfering with viral RNA replication.

### Viruses lacking ExoN-mediated proofreading are more sensitive to treatment with GS-5734.

**FIG 4:**
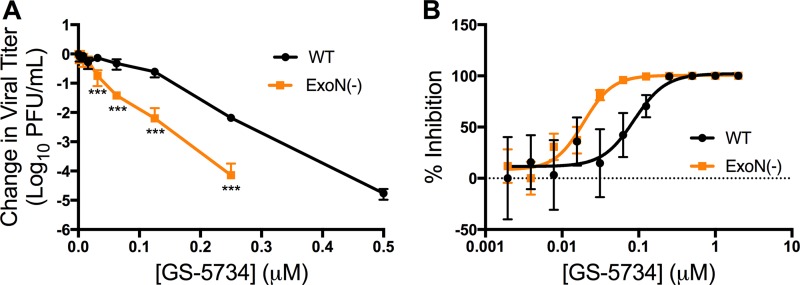
Viruses lacking ExoN-mediated proofreading are more sensitive to GS-5734 inhibition. (A) Change in viral titer of WT and ExoN(−) viruses normalized to vehicle control after treatment with GS-5734. The data represent the results from 2 independent experiments, each with 3 replicates. Error bars represent SEM. Statistical significance compared to WT at each concentration was determined by *t* test using the Holm-Sidak method to correct for multiple comparisons and is denoted by asterisks: ***, *P* < 0.001. (B) Viral titer reduction from panel A represented as percentage of vehicle control, resulting in a WT EC_50_ value of 0.087 μM and an ExoN(−) EC_50_ of 0.019 μM.

We have shown that the profound resistance of CoVs to the nucleoside and base analogues RBV and 5-FU is due to the proofreading ExoN in nsp14, as engineered ExoN(−) mutant MHV and SARS-CoV are profoundly more sensitive to these compounds (22). We therefore compared the sensitivity of WT and ExoN(−) MHV to GS-5734. ExoN(−) MHV demonstrated up to a 100-fold greater reduction in viral titer at 0.25 μM GS-5734 compared to WT virus (Fig. 4A), and the calculated EC_50_ for ExoN(−) virus in this experiment was 0.019 μM, a 4.5-fold decrease compared to the WT EC_50_ of 0.087 μM (Fig. 4B). This increased sensitivity of ExoN(−) virus to GS-5734 is similar to that of other nucleoside analogues and suggests that GS-5734 is incorporated into viral RNA and can be removed by ExoN. However, the results also suggest there is a fundamentally different relationship of GS-5734 with the CoV replicase and/or template RNA compared with other nucleosides such as ribavirin or 5-fluorouracil, since GS-5734 potently inhibits CoVs with intact proofreading (22).

### Two mutations in the RdRp mediate partial resistance and restoration of RNA levels in the presence of GS-5734.

**FIG 5:**
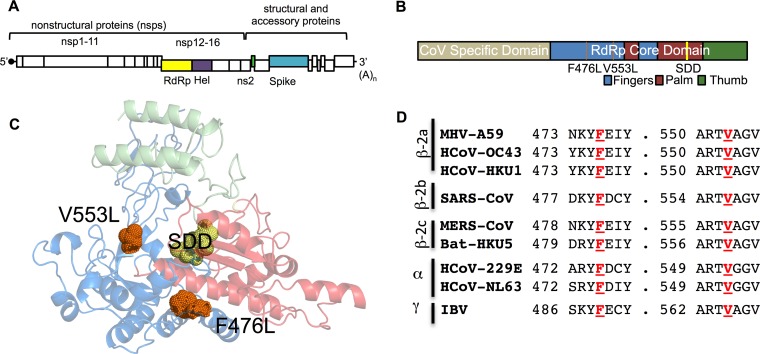
Two mutations in the predicted fingers domain of the nsp12 RdRp, F476L and V553L, arose after 23 passages in the presence of GS-441524, and these residues are completely conserved across CoVs. (A) Schematic of the MHV genome displaying proteins with mutations identified after passage with GS-441524. The nsp12 RdRp is shown in yellow, nsp13-helicase in purple, ns2 in green, and spike in blue. (B) Linear schematic of nsp12 showing the locations of F476L and V553L within the predicted fingers of the RdRp core domain. (C) The previously described (32) Phyre2 model of the MHV RdRp core domain was used to map the predicted locations of the F476L and V553L residues, shown here in orange. The SDD active site residues are shown in yellow, the palm in red, the fingers in blue, and the thumb in green. (D) Amino acid conservation of F476 and V553 residues across CoVs demonstrating that both of these residues are completely conserved.

**FIG 6:**
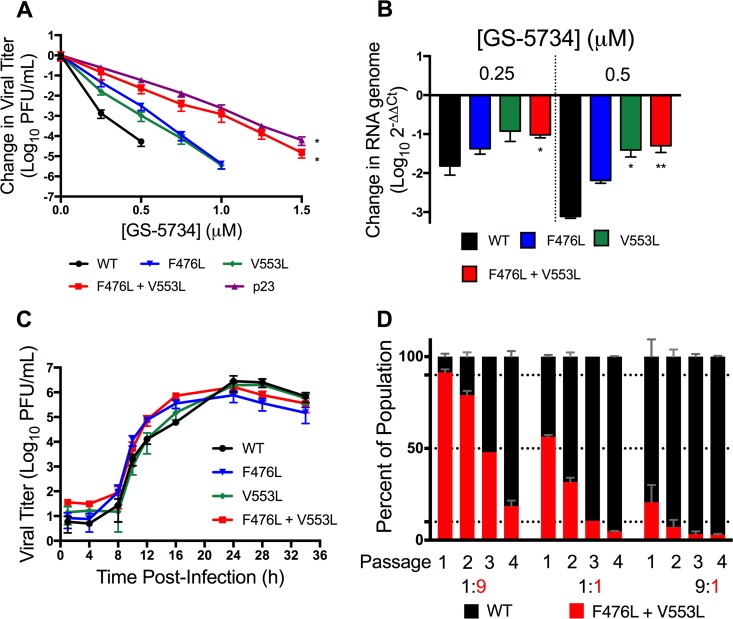
The F476L and V553L mutations mediate resistance to GS-5734 and are associated with a fitness defect. (A) Change in viral titer of WT, F476L, V553L, F476L + V553L, and p23 viruses normalized to the vehicle control after treatment with GS-5734. The data represent 2 independent experiments, each with 3 replicates. Error bars represent SEM. Statistical significance compared to WT was determined by Kolmogorov-Smirnov test and is denoted by asterisks: *, *P* < 0.05. (B) The change in genomic RNA levels of WT, F476L, V553L, and F476L + V553L MHV normalized to vehicle control after treatment with GS-5734. The data represent the results from 2 independent experiments, each with 3 replicates. Error bars represent SEM. Statistical significance compared to WT at each concentration was determined by one-way ANOVA with Dunnett’s *post hoc* test for multiple comparisons and is denoted by asterisks: *, *P* < 0.05; **, *P* < 0.01. (C) Multi-cycle replication kinetics of WT, F476L, V553L, or F476L + V553L MHV. The data represent the results from 2 independent experiments, each with 3 replicates. Error bars represent SEM. (D) Coinfection competition assay of WT and F476L V553L MHV at the indicated ratios. The percentage of the population of each mutation was assessed after four successive passages. The data are representative of 2 independent experiments each with 2 replicates. Error bars represent standard deviation (SD).

We next sought to identify the target(s) of GS-5734 inhibition. Three lineages of WT MHV were serially passaged in the presence of increasing concentrations of GS-441524. GS-441524 was chosen for passage selection because GS-5734 and GS-441524 are both metabolized to the same active triphosphate metabolite (27), but GS-441524 provided a larger working range of concentrations. Two lineages did not demonstrate an increase in viral cytopathic effect (CPE) over passage and were lost after passages 17 (p17) and p20. After 23 passages, we observed an increased ability of one passage lineage to replicate in the presence of GS-441524 as determined by increased viral CPE. Full-genome sequencing of p23 viral RNA revealed 6 nonsynonymous mutations in four viral protein-coding regions (Fig. 5A): the nsp13 helicase (A335V), the ns2 2′,5′ phosphodiesterase (Q67H), the spike glycoprotein (A34V and I924T), and the nsp12 RdRp (F476L and V553L) (Fig. 5B). Molecular modeling of the MHV RdRp predicts that both the F476 and V553 residues reside within the predicted fingers domain of the conserved right-hand structure of the RdRp (Fig. 5C) (32, 33). In addition, both the F476 and V553 residues are identical across sequenced α-, β-, and γ-CoVs (Fig. 5D). Based on the known role of polymerase mutations in resistance to nucleoside analogues for other viruses (34–37) and the previous work describing inhibition of the respiratory syncytial virus (RSV) polymerase by GS-5734 (27), we first engineered and recovered recombinant MHV containing the F476L and V553L RdRp mutations to determine if they were necessary and sufficient for the observed resistance phenotype of the p23 virus population. Recombinant MHV containing either F476L or V553L individually was less sensitive to GS-5734 than WT MHV, but still more sensitive than the p23 virus population across a broad range of concentrations. In contrast, MHV encoding both F476L and V553L demonstrated a resistance pattern comparable to p23 (Fig. 6A). Neither the p23 virus population nor any of the recombinant viruses were completely resistant to GS-5734; all viruses remained sensitive to higher but nontoxic concentrations of GS-5734. Compared to WT MHV, the F476L virus showed 2.4-fold resistance to GS-5734, and V553L virus demonstrated 5-fold resistance to GS-5734, while combined mutations mediated 5.6-fold resistance to GS-5734 based on EC_50_ values (Table 2). Because GS-5734 decreases viral RNA levels, we next tested if resistance mutations restored RNA synthesis. We observed that RdRp resistance mutations partially restored RNA levels in the presence of GS-5734 and that the degree of restoration of RNA levels correlated with their fold resistance to GS-5734 (Fig. 6B). Together, these results are consistent with a mechanism of action of GS-5734 primarily targeting RdRp-mediated RNA synthesis.

**TABLE 2:** F476L and V553L mutations confer up to 5.6-fold resistance to GS-5734 in MHV^a^

Virus	EC_50_ (μM)	Fold resistance
WT	0.024 ± 0.011	1
F476L	0.057 ± 0.040	2.4
V553L	0.12 ± 0.06	5.0
F476L + V553L	0.13 ± 0.06	5.6

a Mean EC_50_ values ± SD and fold resistance of GS-5734-resistant viruses were calculated using viral titer data following infection of DBT cells with the indicated virus at an MOI of 0.01 PFU/cell and treatment with increasing concentrations of GS-5734. Fold resistance was calculated as EC_50_ of mutant/EC_50_ of WT. The data represent the results from 3 independent experiments, each with 3 replicates.

### GS-5734 resistance mutations impair competitive fitness of MHV.

To assess the effect of GS-5734 resistance on viral fitness, we first determined the replication capacity of recombinant MHV carrying the F476L, V553L, and F476L + V553L mutations. Each of these viruses replicated similarly to WT MHV, both in replication kinetics and in observed peak titer (Fig. 6C). We next tested the competitive fitness of F476L + V553L MHV compared to WT MHV during coinfection over multiple passages. Murine DBT cells were coinfected with WT MHV and F476L + V553L MHV at WT/mutant ratios of 1:1, 1:9, or 9:1 in the absence of GS-5734, and infected culture supernatants were serially passaged 3 times to fresh cell monolayers. By passage 2, F476L + V553L MHV was outcompeted by WT MHV in the population at every input ratio (Fig. 6D), demonstrating a competitive fitness cost of the F476L + V553L mutations in the absence of GS-5734. This competitive fitness cost further suggests that GS-5734 resistance mutations will not persist in the absence of treatment.

### Mutations identified in GS-5734-resistant MHV also confer resistance in SARS-CoV.

**FIG 7:**
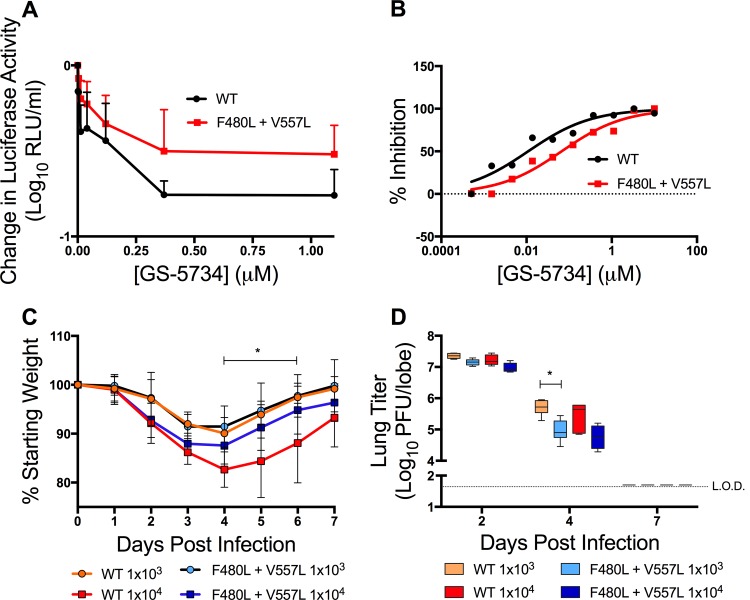
MHV resistance mutations confer resistance and are attenuated in SARS-CoV. (A) Change in luciferase activity normalized to vehicle control of WT or F480L + V557L SARS-CoV containing the NanoLUC reporter. The data are representative of the results from 2 independent experiments, each with 3 replicates. Error bars represent SEM. (B) Viral titer data from panel A presented as the percentage of vehicle control. This EC_50_ value was calculated as 0.01 μM for WT and 0.06 μM for F480L + V557L virus, which represents a 6-fold increase in resistance. (C) Percent starting weight of BALB/c mice inoculated with WT or F480L + V557L SARS-CoV containing the NanoLUC reporter at 10^3^ or 10^4^ PFU. The data are representative of the results from 2 independent experiments, each with 10 to 12 animals per group. Error bars represent SEM. Statistical significance was determined by 2-way ANOVA and is denoted by asterisks: *, *P* < 0.05. (D) Lung titers from animals in panel C 2, 4, and 7 days postinfection. The data are representative of the results from 2 independent experiments, each with 3 animals per group. Error bars represent SEM. Statistical significance was determined by Wilcoxon test and is denoted by asterisks: *, *P* < 0.05.

Given the complete conservation of the F476 and V553 residues across CoVs, we next tested whether substitutions at the homologous SARS-CoV residues (F480L and V557L) could confer resistance to GS-5734. We recovered SARS-CoV carrying the homologous F480L and V557L substitutions and tested recovered mutant viruses for resistance to GS-5734 in Calu-3 2B4 cells. WT SARS-CoV demonstrated dose-dependent inhibition by GS-5734, with an EC_50_ of 0.01 μM (Fig. 7A). The F480L + V557L recombinant virus was inhibited by GS-5734, with an EC_50_value of 0.06 μM, representing a 6-fold resistance to GS-5734 (Fig. 7B), nearly identical to the fold resistance of F476L + V553L MHV. These results support the conclusion that the conserved residues across divergent CoVs reflect conserved functions impaired by GS-5734, potentially implying common pathways to resistance across CoVs.

### GS-5734-resistant SARS-CoV is attenuated *in vivo*.

To gain insight into the pathogenic potential of GS-5734-resistant viruses, we directly compared WT SARS-CoV and F480L V557L SARS-CoV following non-lethal high-dose (10^4^ PFU) and low-dose (10^3^ PFU) inoculation in a well-characterized mouse model of SARS-CoV pathogenesis with disease reminiscent of that observed in humans (38). Mice infected with a high dose of F480L V557L SARS-CoV lost significantly less weight (*P* < 0.05) than WT SARS-CoV-infected mice (Fig. 7C). At 2 days postinfection, mouse lung viral titers were similar between WT and F480L + V557L SARS-CoV, but by 4 days postinfection, lung viral titers were significantly reduced (*P* < 0.05) in mice infected with F480L + V557L SARS-CoV (Fig. 7D). Together, these data demonstrate that GS-5734-resistant SARS-CoV is attenuated in its ability to cause disease and replicates less efficiently than WT virus in robust mouse models of human SARS-CoV disease.

## DISCUSSION

Broadly active antivirals are needed to treat contemporary human CoV infections, including endemic MERS-CoV in the Middle East and potential future zoonotic CoV epidemics. We recently demonstrated the prophylactic and therapeutic efficacy of GS-5734 (remdesivir) in a mouse model of SARS-CoV infection, as well as *in vitro* activity against multiple other human and zoonotic CoVs (23). In this study, we have defined the ability of GS-5734 to inhibit CoVs—expanded to include group 2a β-CoVs—in the setting of intact nsp14 proofreading activities. While ExoN(−) MHV is 4.5-fold more sensitive to GS-5734 treatment than WT MHV, the potent inhibition of WT CoVs suggests a unique mechanism of inhibition of CoV RNA synthesis that is able to circumvent ExoN surveillance and activity. Further, we report for the first time for any virus inhibited by GS-5734 that selection for partial resistance to GS-5734 required prolonged passage. Surprisingly, no resistance mutations were selected within ExoN, but rather two mutations of highly conserved residues in the RdRp reduced the sensitivity to GS-5734 to a level comparable to that of the passaged virus. Introduction of the homologous substitutions in SARS-CoV reproduced the fold resistance to GS-5734 observed in MHV, demonstrating the potential for common, family-wide drug resistance pathways in the RdRp.

### Potential GS-5734 mechanism of action.

Nucleoside analogues can have multiple mechanisms of action, including lethal mutagenesis, obligate or nonobligate chain termination, and perturbation of natural nucleotide triphosphate pools via inhibition of nucleotide biosynthesis (14, 39–44). GS-5734 has been reported to cause premature termination of nascent RNA transcripts by the purified RSV polymerase, but the mechanism of inhibition of other viral polymerases has not been fully explored (27). Our data demonstrate that GS-5734 acts early in infection and decreases RNA levels in a dose-dependent manner that parallels impairment of viral titer. Further, while GS-5734 is highly active against WT CoVs, it is 4.5-fold more active in MHV lacking the proofreading activity of ExoN. Finally, GS-5734 is 3 to 30 times more active than GS-441524 in all of the CoVs we have tested (23). The result is consistent with the report that GS-5734 is metabolized more efficiently than GS-441524 into the triphosphate metabolite (27). All of the above findings support a mechanism involving incorporation of GS-5734 into nascent CoV RNA, but do not discriminate between chain termination and incorporation mutagenesis. In fact, other nucleoside analogues have multiple proposed mechanisms of virus inhibition, including favipiravir in influenza virus and RBV in HCV (41–43). Future studies using deep sequencing and biochemical approaches will allow us to precisely define the GS-5734 mechanism(s) of action against CoVs.

Nucleoside analogues have been approved to treat a variety of RNA and DNA viruses, but CoVs have been refractory to inhibition by some nucleoside analogues (22). This resistance to potent inhibition by RBV and 5-FU has been attributed to the CoV nsp14 proofreading exoribonuclease. We have previously reported that MHV and SARS-CoV strains lacking the proofreading activity of ExoN [ExoN(−)] were more sensitive to 5-FU and RBV, underscoring the role of ExoN-mediated proofreading in resistance to inhibition by these compounds (22). These results suggest that to effectively inhibit CoVs, nucleoside analogues would need to inhibit ExoN directly, be incorporated so efficiently that the 5′-3′ elongation reaction is much faster than the ExoN cleavage reaction, or not be recognized for ExoN-mediated removal. The latter mechanism has been proposed for sensitivity of herpes simplex virus (HSV) to acyclovir; specifically, that the HSV exonuclease is unable to remove acyclovir (45). Here, we show that ExoN(−) MHV is more sensitive than WT MHV to GS-5734 treatment. This result suggests that GS-5734 is recognized, at least partially, by a functional ExoN, but that the ExoN activity is not sufficient to prevent potent inhibition of CoV replication. One possible explanation is that GS-5734 may be recognized and removed by ExoN less efficiently than these mutagens or other incorrect nucleotides, though further studies are needed to fully understand the role of ExoN in GS-5734 inhibition of CoVs. Overall, the enhanced activity of the monophosphate prodrug, the increased sensitivity of ExoN(−) viruses to GS-5734 inhibition, selected resistance mutations in the modeled RdRp finger domain, the time-dependent viral inhibition profile, and decreased viral RNA levels support the hypothesis that GS-5734 directly inhibits viral RNA synthesis.

### Mechanism of resistance to GS-5734.

Previous studies have assessed inhibition by GS-5734 in multiple viruses, but none have reported resistance mutations during treatment. In this study, passage of MHV in the presence of GS-441524 resulted in selection of 5.6-fold resistance. Sequencing identified consensus nonsynonymous F476L and V553L mutations in the nsp12 core polymerase-coding region. A similar level of resistance was observed for the homologous F480L and V557L substitutions in SARS-CoV. As these mutations are not in the immediate vicinity of the RdRp active site, the mechanism of resistance to GS-5734 remains to be determined. Both of these residues are conserved across CoVs, suggesting that they mediate conserved functions. Sequence alignment and molecular modeling of the CoV RdRp predicts that V553L lies within motif F of the fingers domain, which forms a channel for incoming NTPs and contacts the 5′end of the template, while F476L is not within any defined structural motif but also resides in the fingers domain (33, 46). Resistance mutations to nucleoside analogues, including those that lie in the fingers domain, have been implicated in altering replication fidelity as a mechanism of resistance in picornaviruses and HIV (34, 47, 48). In a previous study, using homology modeling of the CoV RdRp based on the coxsackievirus B3 RdRp structure, we predicted and confirmed that a V553I substitution in the MHV RdRp increases CoV fidelity in ExoN(−) viruses (32), suggesting that viral replication fidelity modulation may also impact susceptibility to GS-5734. This is supported by the result that GS-5734, while highly active in WT virus, is even more potent in the absence of nsp14 ExoN proofreading activity. However, the CoV replicase encodes many proteins, and these mutations may alter protein-protein interactions among these components. The availability of an *in vitro* biochemical system demonstrating both polymerase and exoribonuclease activities suggests it may be possible to define specific effects of GS-5734 resistance mutations on polymerase and RNA proofreading activities (49, 50). Thus, it will be interesting to determine if F476L and V553L confer class-level resistance to nucleotide analogues, general increased fidelity, changes in specific nucleotide selectivity, alterations in replicase-protein interactions, or other novel mechanisms.

Recombinant MHV containing the F476L and V553L mutations very closely recapitulated the GS-5734 resistance phenotype of passage 23 virus population, confirming the importance of these mutations for resistance. However, these results do not eliminate the possibility that other potential pathways to GS-5734 resistance may exist. Additionally, we also identified additional non-RdRp mutations in the consensus sequence of passage 23 virus, including another component of the MHV replicase, the nsp13 helicase. It will be important to determine if other proteins contribute to resistance, as well as using GS-5734 as a probe to define protein interactions and functions within the viral replicase.

### GS-5734 resistance is associated with a fitness cost *in vitro* and attenuation *in vivo*.

Identification of resistance mutations to antiviral compound candidates *in vitro* provides an opportunity to assess the concern that resistance may promote viral fitness, leading to enhanced transmission or greater disease severity. The resistance of MHV to GS-5734 was very slow to emerge and only partial, suggesting a high genetic barrier to resistance, similar to that seen for HCV resistance to the nucleotide antiviral sofosbuvir (51). Moreover, although recombinant MHV containing both F476L and V553L replicated similarly to WT in parallel cultures, resistant virus failed to compete with WT MHV during coinfection over multiple passages, demonstrating a fitness cost associated with the resistance mutations that may limit emergence during treatment. The fitness impairment was further evidenced *in vivo* by attenuation of F480L + V557L virus in a SARS-CoV mouse model, similar to that reported for other viruses with selected resistance to nucleotide analogues, including HIV and chikungunya virus (36, 52, 53). This fitness impairment may be due to alterations in RNA replication, fidelity, nucleotide incorporation, or protein stability but suggests that GS-5734 resistance will not lead to more transmissible or pathogenic virus.

In summary, our work provides evidence that GS-5734 is highly active against CoVs and that there is a high genetic barrier to achieve resistance. Additionally, resistant virus suffers a loss of competitive fitness *in vitro* and attenuation in animals, suggesting these mutations will not favor disease emergence and are likely to be poorly maintained in nature, particularly during acute infections. Finally, the results identify potential novel determinants of polymerase function and nucleotide selectivity or fidelity that will guide future structure-function and biochemical studies of the polymerase and GS-5734 mechanism. Together, these results argue strongly for the continued clinical development of GS-5734 to treat MERS-CoV and demonstrate its potential utility in the broad-spectrum treatment of CoV infections.

## MATERIALS AND METHODS

### Cell culture.

Murine astrocytoma delayed brain tumor (DBT) cells and baby hamster kidney 21 cells expressing the MHV receptor (BHK-R) (54) were maintained at 37°C in Dulbecco’s modified Eagle medium (DMEM; Gibco) containing 10% fetal bovine serum (FBS; Invitrogen), penicillin and streptomycin (Gibco), HEPES (Gibco), and amphotericin B (Corning). BHK-R cells were further supplemented with 0.8 mg/ml of G418 (Mediatech). The human lung epithelial cell line Calu-3 (clone 2B4) was kindly donated by C. T. Tseng (University of Texas Medical Branch) (55) and maintained in DMEM (Gibco), 20% fetal bovine serum (HyClone), and 1× Gibco antibiotic-antimycotic solution. Human tracheobronchial epithelial cells were obtained from airway specimens resected from patients undergoing surgery under University of North Carolina Institutional Review Board-approved protocols by the Cystic Fibrosis Center Tissue Culture Core (UNC Tissue Core). Primary cells were expanded to generate passage 1 cells, and passage 2 cells were plated at a density of 250,000 cells per well on Transwell-COL (12-mm-diameter) supports. Human airway epithelium cultures were generated by provision of an air-liquid interface (ALI) for 6 to 8 weeks to form well-differentiated, polarized cultures that resembled *in vivo* pseudostratified mucociliary epithelium (29, 56, 57).

### Viruses.

All work with MHV was performed using the recombinant WT strain MHV-A59 (GenBank accession no. AY910861) (54). SARS-CoV expressing green fluorescent protein (SARS-GFP) and MERS-CoV expressing red fluorescent protein (MERS-RFP) were created from molecular cDNA clones according to protocols described previously (29, 57).

### Compounds and cell viability studies.

GS-441524 and GS-5734 were synthesized at Gilead Sciences, Inc., and prepared as 50 and 20 mM stock solutions in dimethyl sulfoxide (DMSO), respectively. Cell viability was assessed using CellTiter-Glo (Promega) in 96-well plates according to the manufacturer’s instructions. DBT cells were incubated with indicated concentration of compound at 37°C for 24 h. Cell viability was determined using a Veritas microplate luminometer (Promega) with values normalized to those of untreated cells.

### GS-5734 sensitivity studies and generation of EC_50_ curves.

To test MHV sensitivity to GS-441524 and GS-5734, subconfluent monolayers of DBT cells were infected with the indicated virus at a multiplicity of infection (MOI) of 0.01 PFU per cell for 1 h at 37°C. The inoculum was removed and replaced with medium containing the indicated concentrations of GS-441524 or GS-5734. Cell supernatants were harvested 24 h postinfection. Titers were determined by plaque assay (17). To test the GS-5734 resistance capacity of the F480L + V557L SARS-CoV, Calu-3 2B4 cells were seeded in 96-well plates at a density of 5 × 10^5^ cells/well 48 h prior to infection. The medium was replaced with fresh medium 24 h prior to infection to encourage optimal cell growth. Cells were then infected with SARS-CoV F480L + V557L-NanoLuc or SARS-CoV-WT-NanoLuc at an MOI of ~5 PFU/cell in the presence or absence of GS-5734 at 1:3 dilutions, with DMSO (diluent) as an untreated control and UV-inactivated virus as a nanoluciferase reporter (NanoLuc) background control. Cells were lysed after incubation at 37°C for 72 h using a Promega NanoGlo assay kit and assayed on a luminescence plate reader (SpectraMax M3; Molecular Devices). EC_50_ values and curves were generated with the nonlinear regression curve fit in GraphPad Prism software (La Jolla, CA).

### *In vitro* efficacy in human airway epithelial cells.

Fully mature HAE cultures were obtained from the UNC Tissue Core. At 48 h prior to infection the apical surface of the culture was washed with 500 μl 1× phosphate-buffered saline (PBS) for 1.5 h at 37°C, and the cultures were moved into wells containing fresh air-liquid interface (ALI) medium (56). Immediately prior to infection, 500 μl of PBS was added to the apical surface of the HAE cultures for 30 min at 37°C, the first wash was removed, and a second wash was added prior to moving the HAE cultures into ALI medium containing GS-5734 concentrations ranging from 0.0016 to 10 μM, as indicated for each experiment. The second wash was removed, and 200 μl of viral inoculum (MOI of 0.5 PFU/cell for MERS-RFP and SARS-GFP) was added to the apical surface of the cultures for 3 h at 37°C. The viral inoculum was then removed, and the apical surface of the cultures was washed three times with 500 μl 1× PBS, the final wash was removed, and the cultures were incubated at 37°C for a total of 48 h postinfection. For all cultures, apical washes were performed (100 μl 1× PBS) to assess viral replication titers, and then total RNA was collected in 500 μl TRIzol (Life Technologies/ThermoFisher) and frozen at −80°C prior to extraction for real-time PCR analysis. The data that are shown are representative of duplicate sample sets performed with a minimum of three different patient isolates. For the therapeutic HAE experiments, cultures were washed as described above, and HAE cells remained in drug-free ALI medium for the first day of infection. At 24 h postinfection, cultures were moved to ALI medium containing GS-5734 concentrations ranging from 1 to 10 μM as indicated. Cultures were harvested at 48 h post-drug treatment, which was 72 h postinfection.

### Time-of-addition assay.

Subconfluent monolayers of DBT cells were infected with WT MHV at an MOI of 1 PFU/cell for 1 h at 37°C. The virus inoculum was removed, and fresh medium containing DMSO or 2 μM GS-5734 was added at the indicated time postinfection. Supernatant was harvested 12 h postinfection, and the viral titer was determined by plaque assay.

### Real-time qPCR of viral genomic RNA.

Subconfluent DBT cells were infected with the indicated virus at an MOI of 1 PFU/cell (Fig. 3B) or 0.01 PFU/cell (Fig. 6B). The inoculum was removed after 1 h of incubation at 37°C, and medium containing the indicated concentrations of GS-5734 was added. Supernatant was collected, and total RNA was harvested using the TRIzol reagent (Invitrogen) after 10 or 20 h, respectively. The viral titer was determined by plaque assay, and RNA was reverse transcribed using SuperScript III (Invitrogen) to generate cDNA that was quantified by quantitative PCR (qPCR) as previously described (22).

### Selection of GS-5734 resistance mutations.

WT MHV was passaged in triplicate in increasing concentrations of GS-441524, ranging from 1 to 12 μM. Infection was initiated for passage 1 at an MOI of 0.1 PFU/cell. Supernatant was harvested and frozen when the cell monolayer demonstrated 80% cytopathic effect (CPE) or after 24 h. A constant volume of 16 μl was used to initiate subsequent passages. All lineages were maintained until passage 17 (p17). Lineages 2 and 3 were lost after p17 and p20, respectively, when virus CPE did not reach above 50% upon multiple efforts and at various concentrations of GS-441524. Lineage 1 demonstrated an increase in visible CPE, and thus lineage 1 was carried to passage 23. After each passage, total RNA was harvested from infected cell monolayers using the TRIzol reagent to be used for viral population sequencing. After passage 23, RNA was extracted and reverse transcribed using SuperScript III, followed by generation of amplicons for all three lineages covering nsp12 and nsp14 at passage 16 and 12 PCR amplicons to cover the whole genome after 23 passages of lineage 1. Dideoxy amplicon sequencing was performed by GenHunter (Nashville, TN) and analyzed to identify mutations using MacVector.

### Modeling and conservation of resistance mutations in the CoV MHV nsp12 RdRp.

The F476 and V553 residues were located on the previously described MHV RdRp model (32) using the Pymol Molecular Graphics System (Schrödinger, LLC). Multiple sequence alignments were generated using MacVector.

### Cloning, recovery, and verification of mutant viruses.

QuikChange mutagenesis was performed according to the manufacturer’s protocol to generate mutations in MHV individual genome cDNA fragment plasmids using the previously described infectious clone reverse-genetics system (54). Mutants were recovered in BHK-R cells following electroporation of *in vitro*-transcribed genomic RNA. All fragments containing mutations as well as virus stocks were sequenced to ensure mutations were present before use in further studies (GenHunter). To generate SARS-CoV encoding nsp12 resistance substitutions, a 1,450-bp cassette encoding the substitutions (F480L and V557L) was synthesized by BioBasic, Inc. The synthesized cassette was then cloned into the SARS-CoV D infectious cDNA plasmid at unique MluI and MstI sites, and the subsequent selected clone was sequence verified across the cassette. SARS-CoV expressing the resistance substitutions along with the NanoLuc reporter in place of ORF7 was produced as described previously (58).

### Virus replication assays.

Subconfluent monolayers of DBT cells were infected with WT, F476L, V553L, or F476L V553L viruses at an MOI of 0.01 PFU/cell for 1 h. Inocula were removed, and cells were washed with PBS before addition of prewarmed medium. Supernatants were harvested at indicated times postinfection, and titers were determined by plaque assay.

### Competitive fitness of mutant viruses.

Subconfluent DBT cells were coinfected with F476L + V553L and WT MHV at input ratios of 1:9, 1:1, or 9:1 at an MOI of 0.01 PFU/cell for 1 h at 37°C. The virus inoculum was removed, and fresh medium was added. At 20 h postinfection, virus supernatants were collected, and infected cell monolayers were harvested using the TRIzol reagent. Samples were frozen, and cell supernatant was passaged onto fresh DBT cells for a total of four passages. Supernatants and cell monolayers in TRIzol were collected from each passage when nearly all of the monolayer was involved in CPE—approximately 16 h postinfection. RNA was extracted and reverse transcribed using SuperScript III, and PCR amplicons covering the region of the mutations were sequenced (GenHunter). Results represent the combined frequency of F476L and V553L mutations as determined by chromatographic traces and analyzed using MacVector.

### Assessment of resistant virus virulence *in vivo*.

Groups of 10 to 12 10-week old female BALB/c (Charles River, Inc.) mice were anesthetized with ketamine-xylazine and intranasally infected with either 10^4^ or 10^3^ PFU/50 µl wild-type mouse-adapted SARS-CoV expressing nanoluciferase (WT SARS-CoV) or SARS-MA15 NanoLuc engineered to harbor resistance mutations in nsp12 (F480L + V557L SARS-CoV). Animals were weighed daily to monitor virus-associated weight loss. On days 2 and 4 postinfection, 5 to 6 animals per group were sacrificed by isoflurane overdose and the inferior right lobe was harvested and frozen at −80°C until the titer was determined by plaque assay as described previously (38). A 5- to 6-animal cohort was monitored out to 7 days postinfection in order to compare the kinetics of recovery, after which lung samples were harvested and the titer determined as described for previous samples.

### Statistics.

Statistical tests were performed using GraphPad Prism 7 software (La Jolla, CA) as described in the respective figure legends.
